# Density‐Dependent Effects on the Reproductive Ecology of Trees in a Temperate Woodland

**DOI:** 10.1002/ece3.71491

**Published:** 2025-06-02

**Authors:** Eleanor E. Jackson, Matthew P. Greenwell, James M. Bullock, Tom H. Oliver, Susie Topple, Christopher W. Foster, Sofia Gripenberg

**Affiliations:** ^1^ Department of Biology University of Oxford Oxford UK; ^2^ School of Biological Sciences University of Reading Reading UK; ^3^ UK Centre for Ecology & Hydrology Wallingford UK

**Keywords:** density dependence, Hawthorn, Janzen‐Connell hypothesis, pollen limitation, pollination, predispersal seed mortality, Wytham Woods

## Abstract

The reproductive success of plants often depends on their local conspecific densities. The degree of isolation from conspecific plants can mediate an individual's interactions with other organisms. For example, a high density of flowers can attract pollinators and improve seed set, and a high density of seeds can attract enemies such as seed predators. It is the joint outcome of positive and negative density‐dependent effects that will determine the spatial distribution of a population, yet they are rarely studied simultaneously. We related two indicators of reproductive success (fruit set and fruit drop) to tree size and the density of neighbouring conspecifics for 32 *Crataegus monogyna* (Rosaceae) individuals in a temperate woodland. Overall, 26% of flowers set seed, but seed set was not density dependent. We found that 25% of fruits were dropped before reaching maturity, and 24% of mature fruits were dropped before the typical dispersal period. The drop of both immature and mature fruits increased with the density of reproductive conspecifics in this system, with potential implications for spatial patterns of seedling recruitment.

## Graphical abstract

It is the joint outcome of positive and negative density‐dependent effects which will determine the spatial distribution of a plant population, yet they are rarely studied simultaneously. We related two indicators of reproductive success (fruit set and fruit drop) to the size and density of neighbouring conspecifics for *Crataegus monogyna* (Hawthorn) individuals in a temperate woodland. Our results point towards a pattern of net negative conspecific density‐dependence in the pre‐dispersal reproductive success of Hawthorn trees, with individuals in areas with few reproductive conspecifics at an advantage.

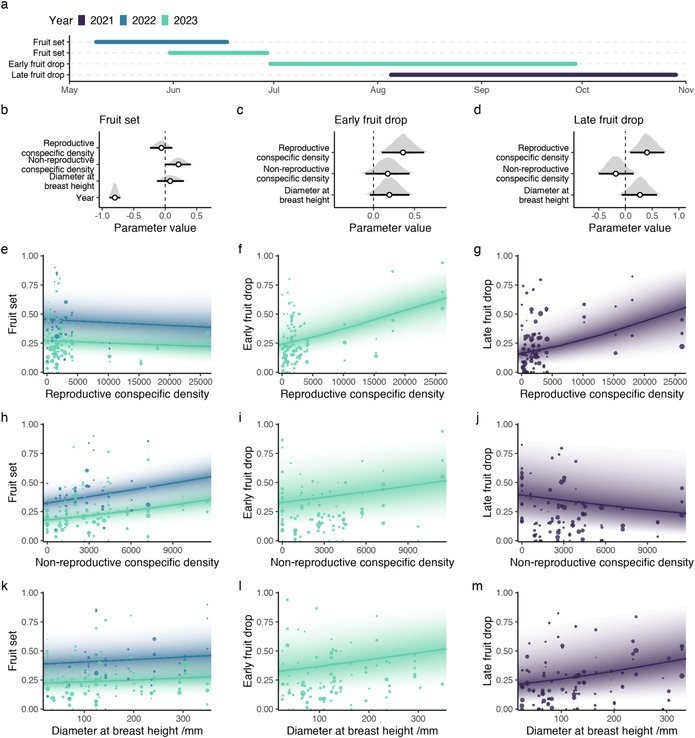

## 1 Introduction

Mutualistic and antagonistic interactions between plants and other organisms are important determinants of plant fitness and can ultimately influence plant demography and community structure (Frederickson & Gordon, Frederickson and Gordon 2007; Harms et al. 2000; Rother et al. 2013). These interactions might be particularly important at the very earliest stages in the life of a plant. Interactions between flowers and pollinators are often crucial for successful reproduction (Karron et al. 2006; Steffan‐Dewenter et al. 2001) and an encounter with a herbivore or seed predator can be fatal for developing seeds (Janzen 1976; Lombardo and McCarthy 2009; Sixtus et al. 2003).

Interactions between plants, their pollinators, and plant enemies are rarely uniform in space (Dupont et al. 2014; Robinson et al. 2023). Larger and denser areas of flowers, whether due to a high local abundance of flowering plants or the large floral display of a single plant, tend to attract more pollinators (Sih and Baltus 1987; Smithson and Macnair 1997). More visits from pollinators benefit the fitness of plants when pollen is limiting, since each pollinator visit can supply more pollen, enabling plants to maximize ovule fertilization. Hence, studies often report a positively density‐dependent pattern in seed set (Dauber et al. 2010; Severns 2003). Higher densities of a plant species can additionally improve seed set by increasing outcrossing. For species with limited seed dispersal distances from the mother tree, neighbouring individuals are often closely related and low seed set in small populations can be reflective of inbreeding depression (Ågren 1996; Hirayama et al. 2007; Severns 2003). However, a high density of developing fruits can also attract plant enemies such as seed predators, especially if they are host‐specific (Östergård and Ehrlén 2005). Such pre‐dispersal attack on reproductive structures by enemies can result in offspring mortality directly through seed predation or indirectly through premature abscission of damaged fruits (fruit drop) (Boucher and Sork 1979; Jackson et al. 2022; Meyer et al. 2014; Planes et al. 2014; Stephenson 1981).

The effects of conspecific density on plant reproduction and fitness have received considerable attention in the context of forest trees. Insect‐pollinated trees and shrubs in isolated forest fragments have been shown to receive less pollen and experience lower levels of outcrossing than individuals in continuous forests, resulting in reduced fruit set for self‐incompatible species (Chacoff et al. 2008; Cunningham 2000; García and Chacoff 2007; Ghazoul and McLeish 2001) and a loss of genetic diversity in the population (Vranckx et al. 2012). Fewer studies have examined pollen limitation in individual trees within forests (but see e.g., Jones and Comita 2008). Although the spatial scale of conspecific isolation between trees within a forest is typically smaller than between trees located in different forest fragments, insect pollinators will often remain foraging in areas of high flower density rather than travel the full distances they are capable of (Goverde et al. 2002; Smithson and Macnair 1997), suggesting that areas of low flower density within forests could still be neglected or less favoured by pollinators.

Another widely documented pattern in forest systems is reduced plant fitness in areas of high conspecific density (conspecific density‐dependence) (Hille Ris Lambers et al. 2002). A potential explanation for this is provided by the Janzen‐Connell hypothesis, which proposes that host‐specific plant enemies reduce plant recruitment and survival in areas of high conspecific density (Connell 1971; Janzen 1970). To date, most studies investigating the effects of conspecific density on tree performance have focused on the survival of dispersed seeds, seedlings, and saplings, with relatively few studies assessing conspecific density‐dependence in the period between successful pollination and seed dispersal (but see Aoyagi et al. 2023; Ballarin et al. 2022; Jones and Comita 2010). This is surprising, given that the reproductive performance of plants can have important effects on both plant populations and communities (Ashman et al. 2004; Turnbull et al. 2000).

In this study, we investigated the effects of tree size and conspecific density on three elements influencing the reproductive success of Hawthorn ( *Crataegus monogyna* ): (1) initial fruit set (the proportion of flowers turning into immature fruits), where higher fruit set could (unless compensated by density‐dependent seed mortality) increase reproductive success, (2) early fruit drop (the proportion of initiated fruits that drop before reaching maturity) and (3) late fruit drop (the proportion of mature fruits that drop *before* the period when the majority of dispersal takes place). (2) and (3) could be triggered by multiple factors operating in a density‐dependent fashion (e.g., inbreeding or attack from natural enemies), but regardless of the mechanism causing fruit drop, the outcome is a decrease in reproductive success. We quantified fruit set and fruit drop on 32 trees distributed across Wytham Woods in Oxfordshire, UK, and related these processes to tree size and conspecific density metrics. Since both pollinators and enemies associated with developing fruits might be attracted to areas of high Hawthorn abundance, we expected large trees and trees in areas with a high density of flowering or fruiting conspecifics to experience both higher initial fruit set and greater fruit drop than smaller trees and trees in areas with a low density of reproductive conspecifics. Additionally, if pollinators and enemies are contributing to density‐dependent fruit set and fruit drop, we expect the density of reproductive conspecifics to have a greater effect on reproductive success than the density of non‐flowering and non‐fruiting conspecifics, since pollinators and enemies would not be attracted to non‐reproductive individuals as food sources or oviposition sites. Specifically, we predict: (a) a positive effect of reproductive conspecific density on fruit set and fruit drop, (b) the effect of reproductive conspecific density on fruit set and fruit drop to be larger than the effect of non‐reproductive conspecific density, and (c) a positive effect of tree size on fruit set and fruit drop.

## 2 Material and Methods

### 2.1 Site and Species Description

We conducted our study between 2021 and 2023 in Wytham Woods, Oxfordshire, UK. The area covers 4.2 km^2^ and comprises a mixture of ancient and secondary woodland alongside plantations from the 19th and 20th centuries (Morecroft et al. 2008).

Hawthorn ( *Crataegus monogyna* ; Rosaceae) is a deciduous small tree or shrub that is common throughout Wytham Woods and across much of Europe (Fichtner and Wissemann 2021; Sorensen 1981). Local densities of Hawthorn vary throughout Wytham Woods (Kirby et al. 2014). Hawthorn is characteristically thorny and densely branched, reaching a height of between 2 and 10 m, with stems reaching 30 cm in diameter for the largest individuals (Fichtner and Wissemann 2021). From April to May, adult Hawthorn produce many thousands of white flowers. Immature green fruits appear in June and ripen from August through September, turning red (Fichtner and Wissemann 2021). Only one seed is produced per fruit. Mature fruits can remain on the plant for over 9 months until they are dispersed by vertebrates, usually birds (Courtney and Manzur 1985; Guitián and Fuentes 1992; Sorensen 1981). A study on the interactions between birds and fruits in Wytham Woods found that most Hawthorn fruits remain on the trees until late November, after which they are consumed by resident and migrating birds and depleted by late December (Sorensen 1981). Hawthorn has self‐incompatible gametes (Raspé and Kohn 2002) and many studies suggest that insects are important for pollination in this species (Chacoff et al. 2008; Fichtner and Wissemann 2021; Guitián and Fuentes 1992; Jacobs et al. 2009; but see Gyan and Woodell 1987b). The white flowers of Hawthorn attract a range of flower visitors, predominantly Diptera but also various Hymenoptera and Coleoptera (Fichtner and Wissemann 2021). Many herbivorous invertebrates are associated with Hawthorn in the UK, including insects that target the reproductive parts of the plant, such as flower weevils (e.g., *Anthonomus pedicularius* and *Anthonomus bituberculatus*), Lepidopteran and Dipteran larvae that consume the fleshy pulp of the fruit (e.g., *Blastodacna hellerella* and *Anomoia purmunda* ), and *Torymus varians* , a hymenopteran seed predator of Hawthorn and apple (Fichtner and Wissemann 2021).

### 2.2 Focal Tree Selection and Estimation of Local Conspecific Density

**FIGURE 1:**
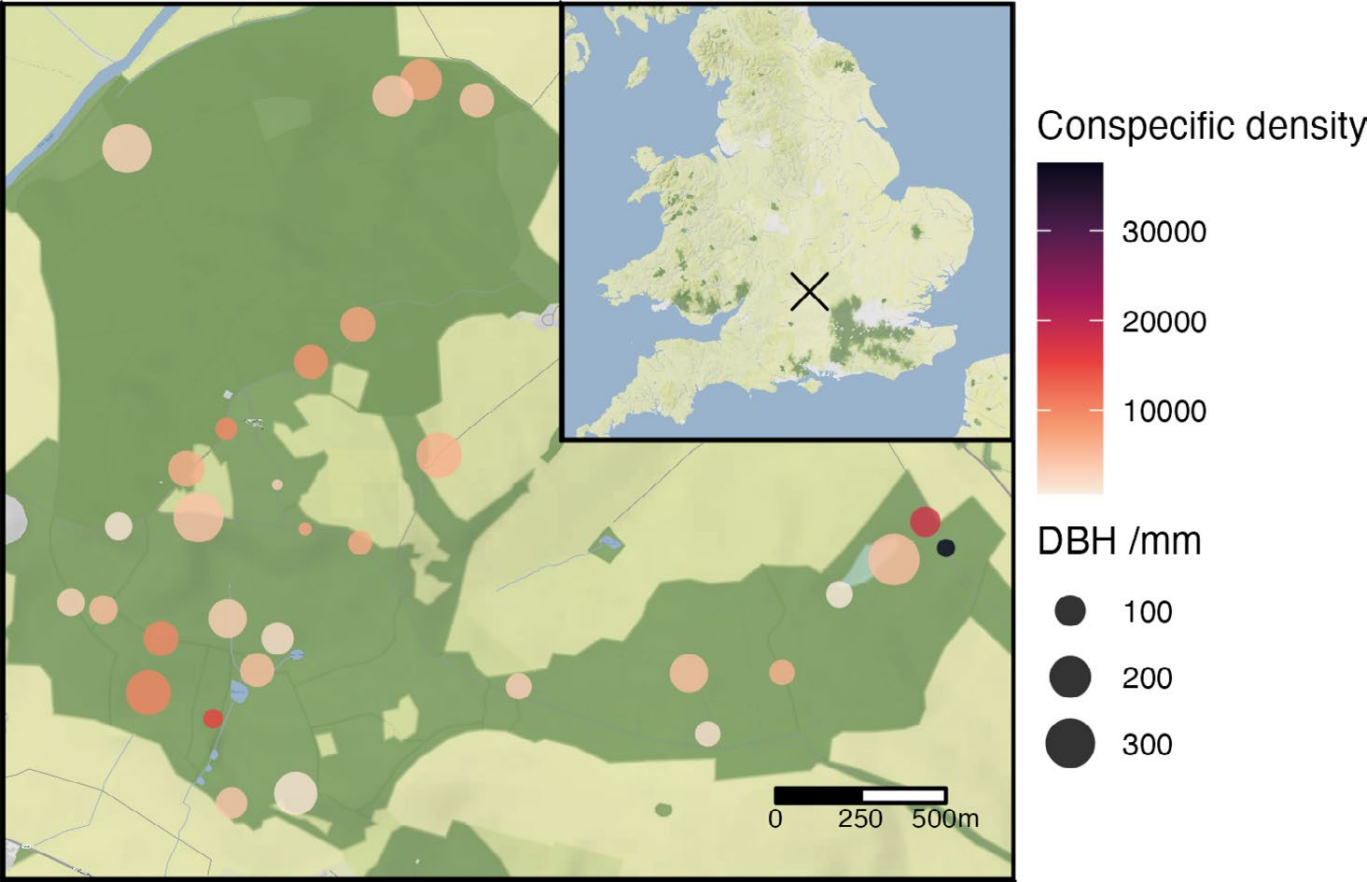
Map of focal Hawthorn trees in Wytham woods. Shaded circles show locations of focal trees with color denoting conspecific density (including both reproductive and non‐reproductive individuals) and size scaled by DBH (*n* = 32). The inset map shows the location of Wytham Woods in the UK. Map tiles by Stamen Design, under CC BY 3.0 and map data by OpenStreetMap under ODbL.

To select Hawthorn individuals for our study, we walked the entire trail network of Wytham Woods in July 2021, opportunistically marking 32 focal study individuals which were easily accessible, reproductive, and ≥ 50 m apart from each other (Figure 1). From May to September 2022, we mapped all Hawthorn individuals within a 50 m radius of each focal tree using a differential global positioning system (dGPS, Emlid Reach RS2+) (see detailed protocol in Appendix). For one of our focal individuals, mapping of conspecifics in the neighbourhood was done in April 2023 since it was missed in the first round of mapping. In addition to coordinates, we recorded the DBH (diameter at breast height of the largest stem) and reproductive status (whether the individual had flowers or fruits at the time of recording) of each mapped individual.

**FIGURE A1:**
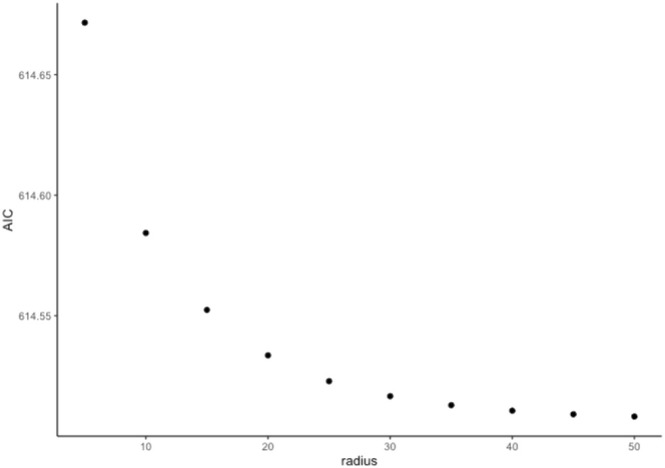
AIC values for models fit with different buffer radii (*r* = 1/α). Binomial general linear models were fit using the R package ‘lme4’. The number of successes was equal to the number of dropped fruits and the number of trials was equal to the total number of fruits. The predictor was total conspecific density which included both reproductive and non‐reproductive conspecific individuals. In this figure, radius is in metres.

We used a size‐ and distance‐weighted conspecific density index (adapted from Hanski 1994) to estimate how connected our focal trees were to conspecifics from the perspective of pollinators or seed predators visiting Hawthorn. The conspecific density index was calculated as:

$$S_{i}=\sum_{j\neq i}\exp\;\left(-\alpha \;{\text{dist}}_{\mathrm{ji}}\right)\;A_{j}$$

where S_*i*_ is the conspecific density at tree *i*, *A*_*j*_ is the DBH (in mm) of conspecific tree *j*, and *dist*_*ji*_ is the distance (in m) from conspecific tree *j* to the focal tree *i*. α is a measure of dispersal propensity (1/average migration distance in m) of a pollinator or seed predator. We chose *α* = 0.02, which corresponds to an average migration distance of 50 m. Since the dispersal distances of insects associated with Hawthorn are unknown and likely to vary across species, we chose this value based on empirical data on the average dispersal distance of a leaf mining moth associated with oak trees (Gripenberg and Roslin 2005) and a study which found pollen limitation in isolated Hawthorn trees at a scale of 50–100 m (García and Chacoff 2007). The maximum possible value of average migration distance was limited by our study design (we only mapped trees within a 50 m radius of our focal trees), but to test if the average migration distance could be less than 50 m, we compared AIC for models fitted with values ranging from 5 to 50 m (Figure A1). It is worth noting that the exact choice of α has a relatively small impact on Hanski's (1994) index (Moilanen and Nieminen 2002). Two measures of conspecific density were calculated for each focal tree: one considering only non‐reproductive conspecifics within 50 m, and one considering only reproductive conspecifics within 50 m. We refer to these as *non‐reproductive conspecific density* and *reproductive conspecific density*, respectively.

### 2.3 Estimation of Fruit Set

**FIGURE 2:**
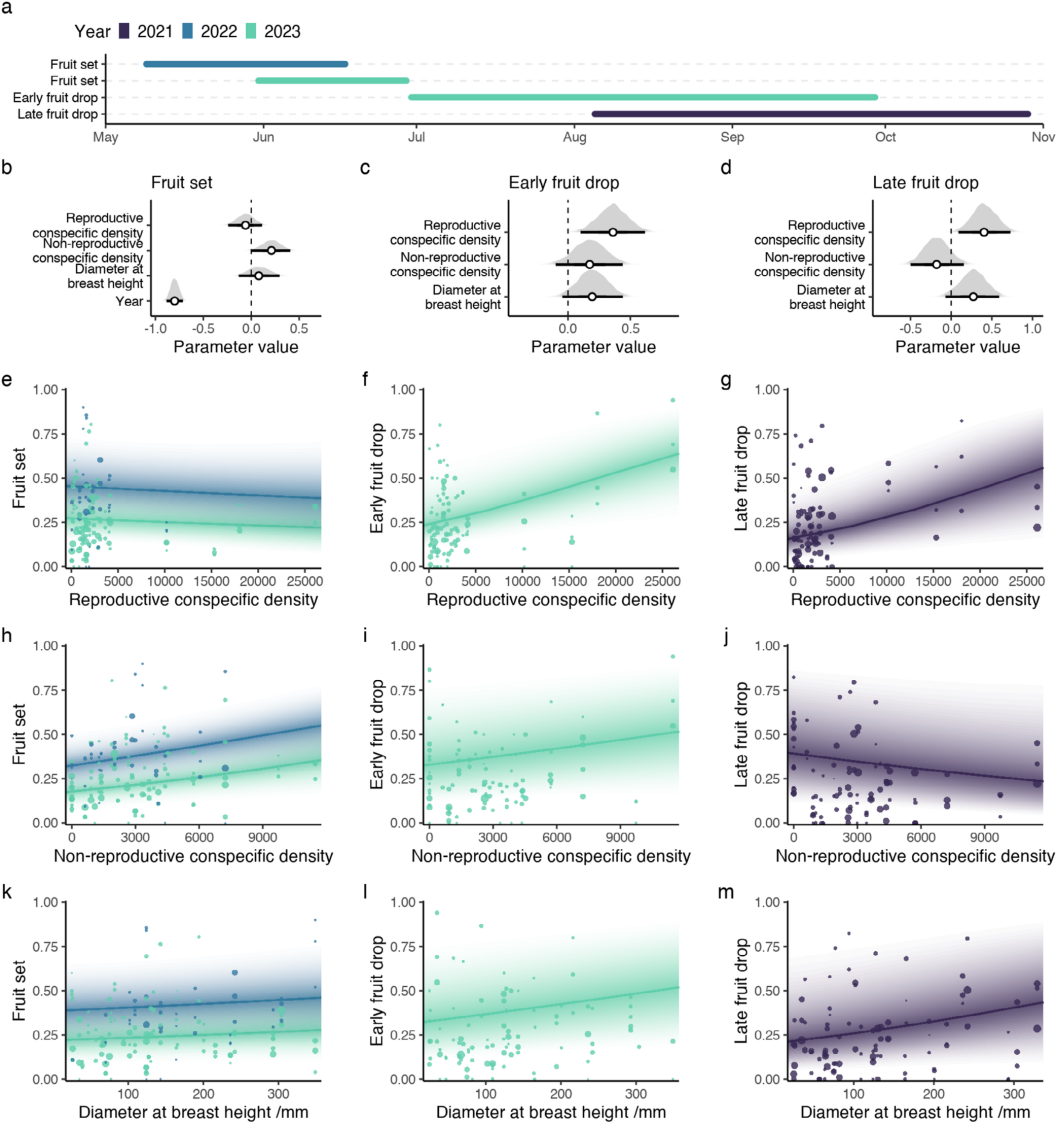
Effects of conspecific density and plant size on Hawthorn reproduction. Panel (a) shows the time period across which each survey was conducted, where colors correspond to year. Panels (b, e, h and k) correspond to the model predicting fruit set, (c, f, i and l) are predictions of fruit drop in the period from initiation to maturity and panels (d, g, j and m) correspond to fruit drop from maturity to the typical dispersal period. Panels (b–d) show the posterior parameter distributions with white points depicting posterior medians and line denotings 95% credible intervals. Panels (e–m) show fruit set for fruit drop plotted against each of their predictors where each point corresponds to a single tree branch with point size scaled by per branch sample size. The solid lines represent the median of all draws from the expected value of the posterior predictive distribution with transparency corresponding to the density of draws.

We estimated fruit set (a measure likely to reflect pollination success) for focal trees in 2022 and 2023 (Figure 2a). To estimate fruit set, we recorded flower abundance on three to six marked branches on each focal tree and returned approximately 1 month later to count immature fruits on the same branches (at this stage fruits were green and not attractive to avian dispersers). As far as possible, we aimed to select branches from different parts of the tree, but for logistic reasons, we had to focus on branches which were accessible from the ground. Branches from the same tree were observed to have similar flower densities (E. E. Jackson, *pers. obs*.). We aimed to survey the same branches in both years; however, when a branch which was reproductive one year did not bear flowers the following year, an alternative branch had to be used. If a marked branch had fewer than 20 flowers and an additional flowering branch was accessible on the same tree, it was also surveyed. In 2022, flowers were counted on 9th, 12th and 13th May, and immature fruits were counted on 16th and 17th June. In 2023, flowers were counted on 31th May and immature fruits on 26th and 29th June. These timings reflect the peak of the flowering period in each year. In 2022, 15 of our 32 focal individuals did not flower; hence, we did not record fruit set for those individuals. In 2023, if a focal individual was not producing flowers (or only very few flowers), but had a reproductive neighbour within 5 m, we estimated fruit set for the neighbouring individual. The neighbouring individual was assumed to have the same conspecific density as the focal tree. We made this substitution for only two individuals (6% of focal trees). The protocol described above resulted in 4187 flowers surveyed across 41 branches from 15 trees in 2022 and 20,525 flowers across 102 branches from 33 trees in 2023.

### 2.4 Estimation of Early Fruit Drop

We estimated early fruit drop for focal trees in 2023 (Figure 2a). On 26th and 29th June 2023, we counted immature fruits on marked branches of each focal tree (same procedure as described above; see ‘Estimation of fruit set’). We surveyed 4812 fruits across 104 branches from 33 trees (32 focal trees plus the approximate nearest reproductive neighbour < 5 m from one of the focal trees). We revisited focal trees soon after fruits turned red, on 29th September 2023, prior to the time period when the majority of Hawthorn seed dispersal by birds takes place (Sorensen 1981) (Figure 2a). The ratio of fruits being dropped versus remaining on each branch between the two surveys was taken as a measure of early fruit drop (see ‘Statistical analysis’ section).

### 2.5 Estimation of Late Fruit Drop

**FIGURE A2:**
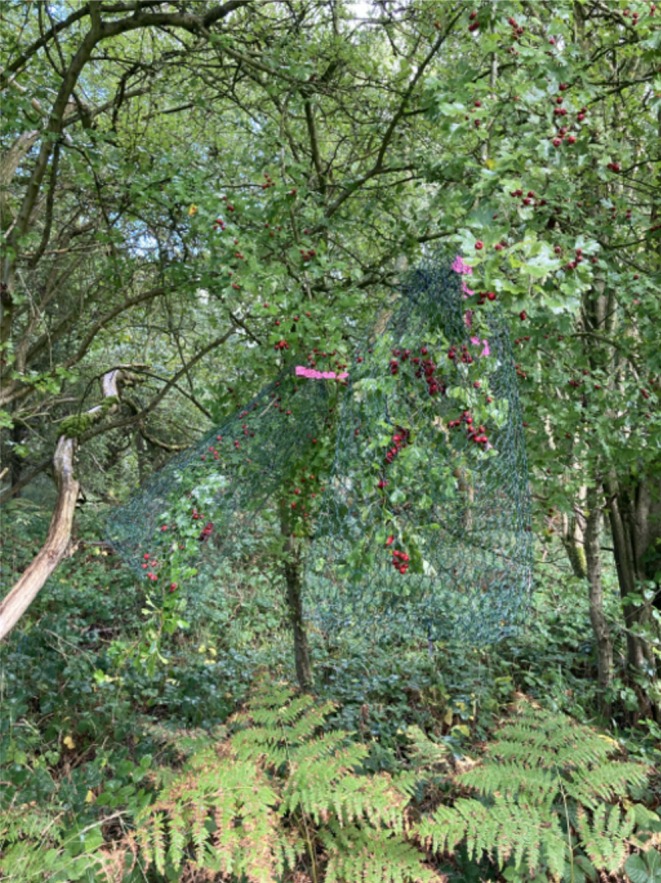
Vertebrate exclusions. Image shows branches of Hawthorn encased in wire mesh to protect fruits from removal by vertebrates.

Late fruit drop was estimated for focal trees in 2021 (Figure 2a). On 5th August 2021, we recorded fruit abundance on three marked branches of each of the 32 focal trees (96 branches with a total of 4989 fruits) and repeated the survey on 29th October 2021. To protect fruits from being removed by vertebrates, marked branches were encased in wire mesh (13 mm mesh size) on 27th, 28th and 29th September 2021 as fruits started to ripen (Figure 2a, Figure A2). The wire mesh size should exclude most vertebrate seed consumers but allow entry to all invertebrate seed predators. In the absence of vertebrate seed consumers, fruit may have remained on the branches for longer than if vertebrates were not excluded, and hence may have been exposed to insects for longer. However, as insect activity is likely diminished during the colder months, when avian consumption is highest, the results from the two methods should be broadly comparable. Additionally, our aim is to assess spatial patterns of insect attack rather than absolute levels of damage. The resulting data sets were used to assess the ratio of fruits dropped versus remaining on the branch between the two surveys.

### 2.6 Statistical Analysis

We analysed the effect of tree size (DBH) and conspecific density (reproductive and non‐reproductive) on fruit set (the proportion of flowers turning into immature fruits) using Bayesian mixed‐effects models with a binomial response distribution estimated using Markov chain Monte Carlo sampling. The number of trials was equal to the number of flowers and the number of successes was equal to the total number of green fruits on a branch. Reproductive and non‐reproductive conspecific density, DBH and year were included as predictor variables (population‐level or fixed effects). Tree ID was included as a group‐level effect (or random effect) to account for the non‐independence of branches within trees.

The effect of tree size (DBH) and conspecific density (reproductive and non‐reproductive) on fruit drop was analysed as above, but with the number of successes equal to the number of dropped fruits and the number of trials equal to the initial total number of fruits. The early and late fruit drop periods were modelled separately. Hence, our analysis consisted of three models in total.

**FIGURE A3:**
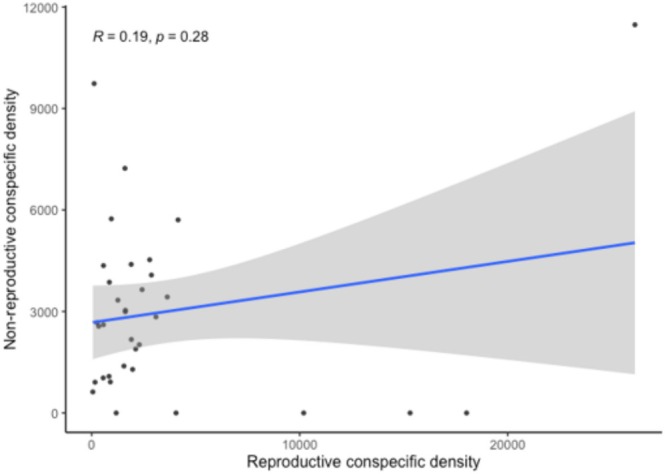
Relationship between reproductive and non‐reproductive conspecific density of Hawthorn. Each point represents an individual tree (*n* = 32) and the solid blue line shows a linear model with confidence intervals of 95%.

**FIGURE A4:**
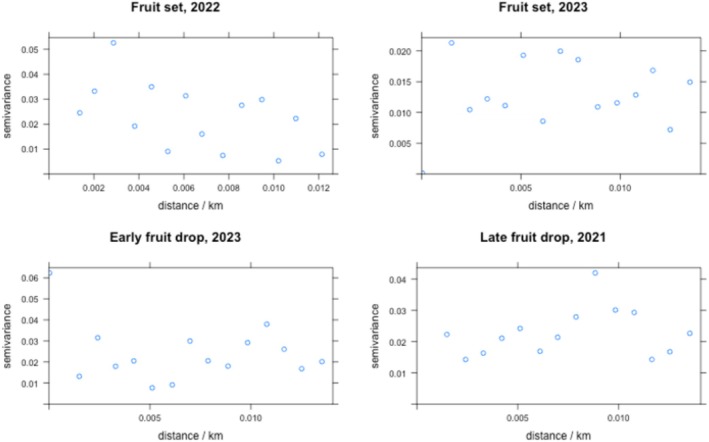
Variograms for fruit drop and fruit set to assess spatial autocorrelation. The x‐axis represents the distance between trees and each point represents a pair of observations. Fruit drop and fruit set are median values per tree.

Before model fitting, we assessed spatial autocorrelation for median values of fruit set and fruit drop per focal tree using variograms created with the R package ‘gstat’ (Gräler et al. 2016; Pebesma 2004). We found no evidence of spatial autocorrelation in fruit set or fruit drop between focal trees (Figure A4). We also assessed the relationship between reproductive and non‐reproductive conspecific density and found no correlation (*r* < 0.2, *p =* 0.28, Figure A3). To facilitate model convergence and interpretation of posterior parameter estimates, we scaled all predictor variables to a mean of zero and variance equal to one standard deviation in all models. The population‐level effects were given weakly informative normal priors with a mean of zero and standard deviation of one. For the population‐level intercept and the group‐level effect of tree ID we used the default priors provided by the R package ‘brms’ (Bürkner 2017): a half student‐t prior with a location of zero, three degrees of freedom and scale parameter of 2.5.

**FIGURE A5:**
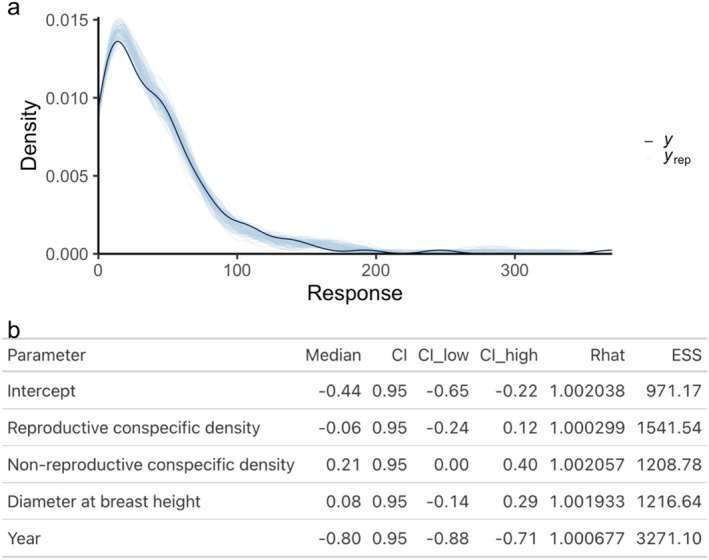
Supplementary model outputs for prediction of fruit set. Panel (a) shows a posterior predictive check where the observed data is in dark blue (*y*) and simulated samples from the posterior distribution are in light blue (*y*_*rep*_). Panel (b) is a table of model output which includes: Posterior medians, 95% credible intervals, $\hat{R}$ and Effective Sample Size (ESS) for population‐level parameters in the model.

**FIGURE A6:**
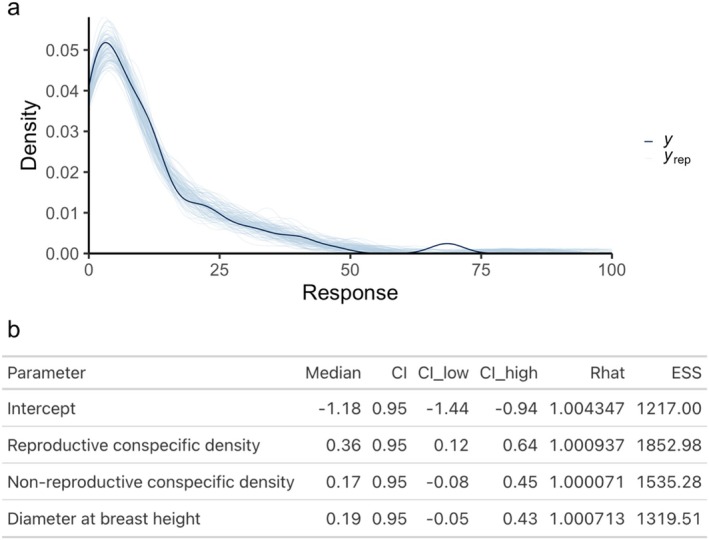
Supplementary model outputs for prediction of early fruit drop. Panel (a) shows a posterior predictive check where the observed data is in dark blue (*y*) and simulated samples from the posterior distribution are in light blue (*y*_*rep*_). Panels (b) is a table of model output which includes: Posterior medians, 95% credible intervals, $\hat{R}$ and Effective Sample Size (ESS) for population‐level parameters in the model.

**FIGURE A7:**
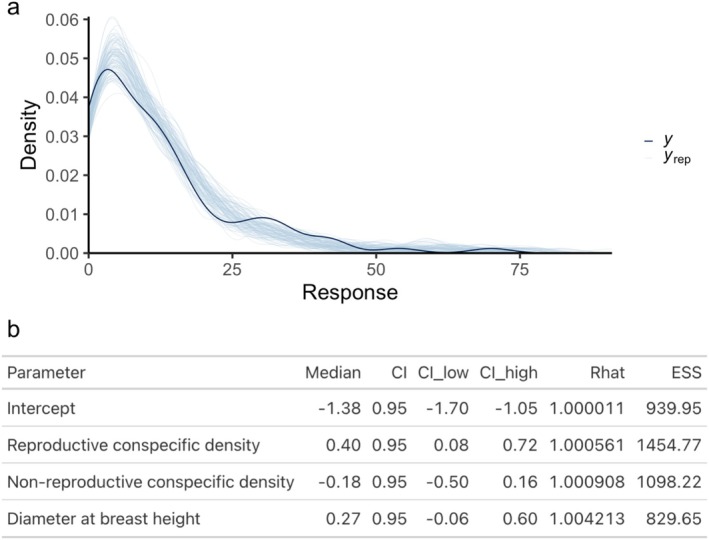
Supplementary model outputs for prediction of late fruit drop. Panel (a) shows a posterior predictive check where the observed data is in dark blue (*y*) and simulated samples from the posterior distribution are in light blue (*y*_*rep*_). Panel (b) is a table of model output which includes: Posterior medians, 95% credible intervals, $\hat{R}$ and Effective Sample Size (ESS) for population‐level parameters in the model.

All analyses were conducted in R (version 4.2.3) (R Core Team 2023). The Bayesian models were implemented in Stan (Carpenter et al. 2017) and run in R through the R package ‘brms’ (version 2.19.0) (Bürkner 2017). For each of the models, we ran four parallel chains. The initial values for the chains were chosen at random within a reasonable range, as is default in ‘brms’. Each chain was run for 2000 iterations with a warm‐up phase of 1000 iterations. We checked for convergence by visually inspecting the chains and verifying that $\hat{R}$ was below 1.01 for all parameters of the fitted model (Figures A5, A6, A7).

We used posterior predictive checks to assess the global fit of the model to the data (Figures A5, A6, A7). We present median estimates along with 95% credible intervals (Highest Density Interval) for all estimates as measures of uncertainty. The probability of direction is reported as an index of effect existence (i.e., the percentage of the posterior distribution which is of the median's sign). We consider the reported effect to have a non‐negligible or “significant” change on the outcome when the 95% credible intervals do not contain zero.

## 3 Results

Overall, 26% of sampled flowers turned into immature fruits, 25% of immature fruits were dropped before reaching maturity, and 24% of mature fruits were dropped before the typical dispersal period. In other words, on average only 15% of flowers resulted in a mature fruit.

Fruit set (the proportion of flowers turning into immature fruits) had negligible relationships with tree size, reproductive, and non‐reproductive conspecific density. The effect of reproductive conspecific density was slightly negative but negligible (75% probability of being negative, median = −0.06, 95% CI [−0.24, 0.12]) (Figure 2b,e and Figure A5). The effect of non‐reproductive conspecific density on fruit set had a 98% probability of being positive (median = 0.21, CI [0.00, 0.40]) but would still be considered non‐significant with our chosen threshold of 95% credible intervals (Figure 2b,h and Figure A5). The effect of tree size (DBH) on fruit set was small and negligible (77% probability of being positive, median = 0.08, CI [−0.14, 0.29]) (Figure 2b,k and Figure A5). Fruit set, however, was strongly associated with year (median = −0.80, CI [−0.88, −0.71]), with a significantly higher proportion of flowers turning into fruits in 2022 than in 2023 (Figure 2b,e,h,k and Figure A5).

Both the proportion of immature fruits that were lost from branches (early fruit drop in 2023) and the proportion of mature fruits lost before the typical dispersal period (late fruit drop in 2021) increased significantly with reproductive conspecific density. The early and late periods of fruit drop both demonstrated negligible relationships with non‐reproductive conspecific density and tree size. Tree size had a negligible positive effect on early fruit drop (95% probability of being positive, median = 0.19, CI [−0.05, 0.43]) (Figure 2c,l and Figure A6) and late fruit drop (94% probability of being positive, median = 0.27, CI [−0.06, 0.60]) (Figure 2d,m and Figure A7). The effect of reproductive conspecific density was significantly positive for both early fruit drop (99.5% probability of being positive, median = 0.36, CI [0.12, 0.64]) (Figure 2c,f and Figure A6) and late fruit drop (99.3% probability of being positive, median = 0.40, CI [0.08, 0.72]) (Figure 2d,g and Figure A7). The effect of non‐reproductive conspecific density on early fruit drop was positive but negligible (91% probability of being positive, median = 0.17, CI [−0.08, 0.45]) (Figure 2c,i and Figure A6), whereas the effect of non‐reproductive conspecific density on late fruit drop was negative but negligible (87% probability of being negative, median = −0.18, CI [−0.50, 0.16]) (Figure 2d,j and Figure A7).

## 4 Discussion

Our study of the reproductive ecology of Hawthorn in Wytham Woods provides evidence to support a negative effect of conspecific density on reproductive success. We found that rates of flower to fruit transition (fruit set) did not correlate with the density of conspecifics. However, in areas with a high local density of fruiting conspecifics, fruits were less likely to survive to maturity and less likely to remain on the tree until the period when the majority of dispersal takes place. Below we discuss some potential explanations for and implications of these findings.

### 4.1 Fruit Set

On average, we found that 26% of an individual's flowers gave rise to green, immature fruits. A flower‐to‐fruit conversion rate of 26% agrees with reported rates of fruit set from Hawthorn populations in northern Spain, which vary between 20% and 50% under natural conditions (Chacoff et al. 2008; Guitián and Fuentes 1992). Fruit set was consistently higher in 2022 compared to 2023, likely due to reduced flower production in 2022 (observed, but not directly comparable with our data since different branches were surveyed). Reduced flower production in 2022 might be attributable to springtime drought experienced in the UK that year (March—May), where rainfall was 70% below average across most regions, including our study site (Barker et al. 2024).

We expected flower‐to‐fruit transition rates to be highest in areas with many Hawthorn flowers, whether in the form of many flowering individuals or large individuals with many flowers. Areas of high floral density attract pollinators (Sih and Baltus 1987; Smithson and Macnair 1997) and seed set generally increases with increased pollinator visits when pollen is limiting (Aizen and Harder 2007; Ashman et al. 2004). A high density of conspecific individuals can additionally increase seed set through qualitative mechanisms, as the number of high‐quality pollen donors in the neighbourhood may be greater (Aizen and Harder 2007). However, we found no support for our hypothesis in our study population, with both focal tree size and reproductive conspecific density having small, negligible effects on fruit set. Surprisingly, we did see a positive but marginal effect of non‐reproductive conspecific density on fruit set. Since non‐flowering trees would not be attractive to pollinators, perhaps this result is reflective of unmeasured variables that could correlate with both conspecific density and fruit set; for example, favourable abiotic conditions. The lack of density dependence in fruit set contrasts with a previous study on Hawthorn populations (García and Chacoff 2007) and a number of studies conducted in other systems (Aizen and Harder 2007; Augspurger 1980; Ballarin et al. 2022; Ghazoul 2005; Grindeland et al. 2005; Sih and Baltus 1987). While Hawthorn is generally reported as being reliant on pollinators for fertilization (Chacoff et al. 2008; Fichtner and Wissemann 2021; Guitián and Fuentes 1992; Jacobs et al. 2009), a previous study conducted in Wytham Woods concluded that the *Crataegus monogyna* population was autogamous (Gyan and Woodell 1987b). If this is indeed true, it could explain why we did not find the expected pattern between the density of flowering conspecifics and fruit set. There are also other potential explanations for the flat relationship between fruit set and reproductive conspecific density in this study. Where flowers are abundant, it is possible that flower‐visiting insects cannot ‘keep up’ with a high resource abundance (e.g., Menge et al. 2017). Moreover, if pollinators forage over relatively large areas, even the most isolated Hawthorn individuals in our study may have been highly accessible to pollinators. It could also be possible that the broad patterns of host use shown by many pollinators in the UK (e.g., Memmott 1999) will make conspecific density effects less likely, as pollinators are recruited from other flowering species. However, many of the insects visiting Hawthorn flowers could be considered functional specialists, since at Wytham Woods, Hawthorn is likely to be the most abundant floral resource at the time of flowering (Gyan and Woodell 1987a, 1987b). Blackthorn ( *Prunus spinosa* ; Rosaceae) shares a similar flower morphology and is attractive to a similar range of pollinators as Hawthorn, but the two species were previously shown to have sharply differentiated flowering phenology at our study site (Gyan and Woodell 1987b).

Regardless of whether pollinator visits were higher in areas of high Hawthorn flower density, we might have expected to see some evidence of *qualitative* pollen limitation. García and Chacoff's (2007) study found a correlation between fruit set and canopy cover (correlated with Hawthorn density at their site) in fragmented Hawthorn populations. However, the rate of insect visitation did not change with canopy cover, suggesting that the pattern of low fruit set in isolated individuals was driven by qualitative mechanisms. Increased distances between conspecific trees can result in increased self‐pollination, as pollinators stay longer and visit more flowers per plant when visiting isolated individuals (Klinkhamer and de Jong 1990; Schulke and Waser 2001). Despite García and Chacoff's (2007) study and the current study being conducted at similar spatial scales, it is possible that some element of pollinator foraging behaviour makes outcrossing less likely for isolated individuals in matrix habitat compared to isolated individuals in continuous forest.

**FIGURE A8:**
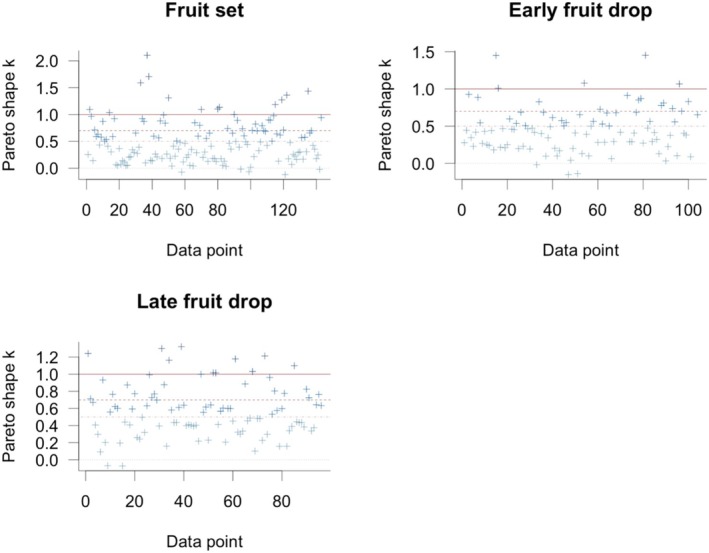
Estimated Pareto shape parameters for each model to assess the influence of observations on model fit. Figures, created with the R package 'loo}, show the estimated shape parameter *k* for each observation, which can be used as a measure of the observation's influence on the posterior distribution of the model. Highly influential observations have high Pareto *k* values (above 0.7) (Vehtari et al. 2017, 2024). Each panel corresponds to one of our three fitted models.

Finally, we note that due to logistical constraints (primarily the workload involved in ground‐based mapping of Hawthorn individuals in the neighbourhood of focal trees), the number of sampled Hawthorn individuals in our study was relatively small. Nevertheless, the fact that we did pick up density‐dependent effects in terms of fruit drop in the same study system suggests that it is unlikely we will have missed any strong effects of conspecific density on fruit set due to limited statistical power. We also note that our study included few individuals in areas with a high density of reproductive conspecifics. This is reflective of the spatial distribution of Hawthorn in Wytham Woods. Very high‐density clusters of Hawthorn generally contained smaller non‐reproductive individuals. Low sampling at the extreme end of reproductive conspecific density could lend disproportional leverage to these outlying values (Figure A8).

### 4.2 Fruit Drop

A similar mean proportion of fruits was lost in the partially overlapping early (2023) and late (2021) fruit drop periods (25% and 24% respectively), implying that at least a quarter of initiated fruits are lost before the typical dispersal period. As predicted, trees in areas with a high density of reproductive conspecifics dropped more of their fruits, in both the early and late fruit drop periods. This is in contrast to the local density of non‐reproductive conspecifics, which did not have a significant effect on early or late fruit drop in our focal trees. We also found positive but marginal effects of tree size on fruit drop in both time periods, providing tentative support for the prediction that larger trees tend to drop a higher proportion of their fruits prior to dispersal.

**FIGURE A9:**
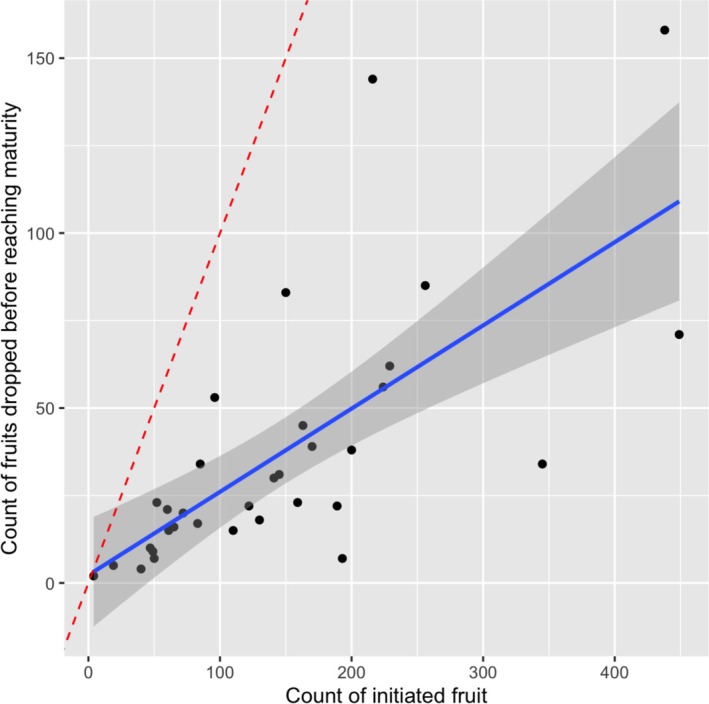
Relationship between the count of initiated fruits in 2023 and the proportion of those which had dropped from the tree before reaching maturity. Counts are summed across branches for each individual Hawthorn tree (*n* = 32). The dashed red line indicates where x = y and the solid blue line shows a linear model with confidence intervals of 95%.

Fruit drop can be triggered by damage to the fruit or seed (caused by enemy attack or abiotic factors such as storm damage), or through selective abscission of undamaged fruits by the parent tree (Stephenson 1981). Abiotic damage to fruits, while a potential cause of fruit drop, is unlikely to occur in density‐dependent patterns. Selective abscission is often due to limiting resources or pollination failure, and usually happens soon after flowering (Tromp and Wertheim 2005). We found that early fruit drop increased proportionally with the number of fruits initiated (Figure A9), such that for any number of initiated fruits, a plant is expected to drop 25% before fruit maturity. This is characteristic of fruit abortion driven by limited resources (Stephenson 1981) and supports the theory that young fruits compete for limited resources in our studied individuals. Resource availability and pollination success are likely to vary across space and could have contributed to fruit losses in the early fruit drop period before fruits reached maturity. We detected no correlation between fruit set and reproductive conspecific density (which would be expected if pollinators were attracted to areas of high flower density) and since density‐dependent pollination would predict the opposite effect of conspecific density on early fruit drop than is observed here, it is unlikely that pollination failure contributed to density‐dependent patterns of early fruit drop described in this study.

It is unlikely that fruit drop due to resource limitation or pollination failure was captured during the late period of fruit drop, since fruits were already mature. Whilst we did not quantify enemy attack and its potential link to fruit drop (this would have required us to sample fruits from the studied branches which could have affected rates of abscission of remaining fruits), it seems plausible that some fruit drop may have been caused by insect fruit and seed predators. Adult *Anomoia purmunda* (Diptera: Tephritidae), a Hawthorn‐specific fruit predator, was observed at focal trees and we found many fruits infested with dipteran and lepidopteran larvae (likely Hawthorn specific *Blastodacna hellerella* as reported by Manzur and Courtney (1984)). Other studies in Europe have measured infestation of *Anomoia purmunda* in Hawthorn fruits at 52% and 40% (Guitián and Fuentes 1992; Teodoru et al. 2015), Lepidopteran larvae infestation at 22% (Guitián and Fuentes 1992), and overall insect damage at 37% (Courtney and Manzur 1985). Tephritid flies have been reported to cause fruit drop in many agriculturally important crops (Dutta et al. 2022; Stonehouse et al. 2002), so it seems plausible that some of the fruit drop observed in our study could indeed have been triggered by Tephritid flies. Attack by specialist natural enemies is usually highest where their host plant is most abundant, since high‐density areas of individual plants, seeds, or fruits are likely easier to locate and more attractive to dispersing enemies (Castagneyrol et al. 2013; Gripenberg and Roslin 2005). The stage at which natural enemies attack Hawthorn fruits is unknown. Tephritid flies in other systems have exhibited preference for ripe fruits as oviposition sites, but lay eggs on green fruits nonetheless (albeit fewer) (Jang and Light 1991). Hence, it is possible that enemy attack is partly responsible for density‐dependent patterns of fruit drop in both the immature and mature fruit stages. Whilst fruits dropped due to insect attack could still contain viable seeds, the probability that they will germinate and mature is low. Birds often reject insect‐infested Hawthorn fruits making dispersal unlikely (Manzur and Courtney 1984) and seeds which remain under the canopy of their parent tree may experience increased rates of attack from enemies and higher intraspecific competition if they were to successfully germinate (Howe 1989; Murphy et al. 2017).

## 5 Conclusions

We aimed to understand the spatial density‐dependence of tree reproductive success in a temperate woodland. Despite reproductive success being a function of positive and negative processes, they are rarely studied simultaneously. While it was not possible to follow the same cohorts of flowers and fruits through to maturation in this study, our results point towards a pattern of net negative effects of conspecific density in the pre‐dispersal reproductive success of Hawthorn trees, with individuals in areas with few reproductive conspecifics at an advantage. Although it would be premature to draw firm conclusions on the broader implications of the patterns documented in our study, they align with predictions from the Janzen‐Connell hypothesis whereby specialist plant enemies promote plant species coexistence through conspecific density‐dependence (Connell 1971; Janzen 1970). While the joint outcome of positive and negative density dependent processes is what will ultimately influence species' distributions in space, understanding how different components (e.g., enhanced pollination versus higher pressure from enemies under high local densities) shape patterns of recruitment will give us a better understanding of spatial population processes of potential key relevance for diversity maintenance in plant communities and the likely implications of potential perturbations in these processes.

## Author Contributions

**Eleanor E. Jackson:** conceptualization (equal), data curation (lead), formal analysis (lead), funding acquisition (equal), investigation (lead), methodology (lead), project administration (lead), software (lead), visualization (lead), writing – original draft (lead), writing – review and editing (equal). **Matthew P. Greenwell:** data curation (supporting), investigation (equal), methodology (supporting), project administration (supporting), writing – review and editing (equal). **James M. Bullock:** conceptualization (supporting), methodology (supporting), supervision (supporting), writing – review and editing (equal). **Tom H. Oliver:** conceptualization (supporting), methodology (supporting), supervision (supporting), writing – review and editing (equal). **Susie Topple:** investigation (supporting), methodology (supporting), writing – review and editing (equal). **Christopher W. Foster:** investigation (supporting), methodology (supporting), supervision (supporting), writing – review and editing (equal). **Sofia Gripenberg:** conceptualization (equal), funding acquisition (equal), investigation (supporting), methodology (supporting), supervision (lead), writing – review and editing (equal).

## Conflicts of Interest

The authors declare no conflicts of interest.

## Data Availability

Data is archived at https://doi.org/10.5281/zenodo.10599206 (Jackson et al. 2024) and code is available at https://github.com/ee‐jackson/wytham‐hawthorn and archived at https://doi.org/10.5281/zenodo.15124592 (Jackson 2025).

## Appendix A dGPS Mapping Protocol

From May 2022 to September 2022, the locations of all observed Hawthorn stems were recorded within a 50 m radius of each of the focal individuals using a differential global positioning system (dGPS, Emlid Reach RS2+). The RS2+ provides three levels of accuracy, which vary depending on multiple factors, including the number of satellites available and the sky view around points. We only recorded location points (latitude and longitude) when the dGPS was recording in one of the two highest accuracy modes (FLOAT and FIX), providing sub‐metre and cm level accuracy, respectively.

For each focal tree, we measured the diameter at breast height (DBH, where BH = 1.3 m) of all established stems with a DBH greater than 10 mm within the 50 m radius, following Condit (1998). Stems with DBH < 10 mm were classified as saplings and had their location recorded, but DBH was not measured. Established (non‐sapling) trees were classified as either single stem or multiple stem trees. Where trees had multiple stems, the DBH of each stem was recorded. Where single stems divided from a main stem into multiple, secondary stems, the height at which the division occurred on the main stem was recorded, and the diameter was measured at that height. The DBH of each secondary stem was also recorded. We also noted whether stems were dead, leaning or horizontal, or if they were broken above or below breast height.

In addition to coordinates and DBH, we recorded the reproductive status (whether the individual had flowers or fruits at the time of recording) of each individual.
